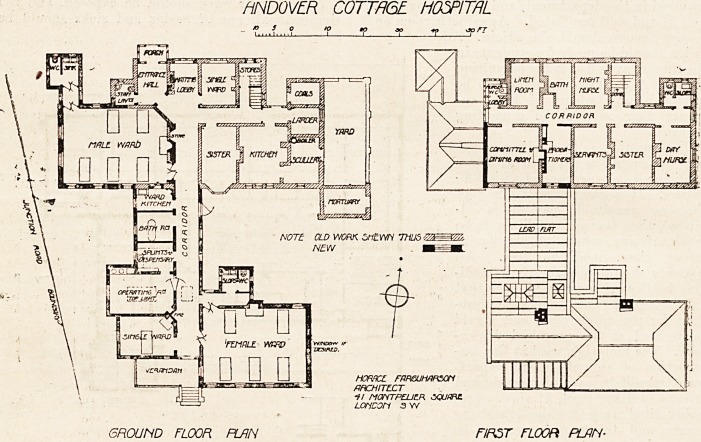# Andover Cottage Hospital

**Published:** 1907-06-29

**Authors:** 


### ANDOVER COTTAGE HOSPITAL.

This hospital, as it was before the alterations, consisted of a two-story building, with the wards, three in number, on the upper floor—a curious arrangement, which must have entailed a quite unnecessary amount of labour in carrying helpless patients upstairs.

In the new work the old wards have been converted to administrative purposes, and new wards, with their offices erected on the ground floor.

The extent of the site not being shown on the plan, it is somewhat difficult to appreciate the difficulties which beset without the necessity of passing through the large ward—a very reasonable requirement, but one which might have been met in another way. The kind of plan which suggests itself, assuming there were no difficulties in the matter of site, is to make the old building a central administration block, and to place the wards north and south. If some such plan as this could have been contrived, the hospital would, we think, have been more compact and easier of administration generally. The position of the operation theatre, with an aspect a little south of west, is unfortunate,

GROUND FLOOR PLAN. FIRST FLOOR PLAN.

the architect in planning the new wards. Neither of the wards can be said to be entirely satisfactory, as in each case a substantial part of one side-wall is blocked for ventilation purposes by adjoining buildings. In the case of the male ward the boundary of the site prevents the ward being pushed further out; while in the case of the female ward the position of the sanitary offices effectually prevents cross-ventilation on the greater part of the north side. In the latter case the committee, we understand, required that the sanitary offices should be available for the one-bed ward but it is difficult to see where it could have been placed so as to get a north aspect. But the central skylight is a mistake that ought to have been avoided. An architect having to plan an operation theatre ought to know that the very best form of light is a vertical window, with a skylight in one continuous line. It is not only the best arrangement as regards light, but it economises space in the theatre. The architect was Mr. Horace Farquharson, of Knightsbridge; the contractors were Messrs. Beale and Sons, of Andover. The cost of the alterations has amounted to a little over £1,400.

## Figures and Tables

**Figure f1:**